# Depression, Depression Treatments, and Risk of Incident Dementia: A Prospective Cohort Study of 354,313 Participants

**DOI:** 10.1002/alz.084340

**Published:** 2025-01-03

**Authors:** Liu Yang, Jin‐Tai Yu

**Affiliations:** ^1^ Fudan University, Shanghai, Shanghai China; ^2^ Huashan Hospital, Fudan University, Shanghai, Shanghai China

## Abstract

**Background:**

To investigate the associations between courses of depression, the application of depression treatment, and the risk of incident dementia.

**Method:**

In this prospective cohort study, 354,313 participants aged 50 to 70 years were recruited from the UK Biobank between 2006 and 2010, and were followed‐up until 2020, with a total of 4,212,929 person‐years. We initially studied the effect of depression on dementia incidence across four subgroups characterized by courses of depressive symptoms. Then, 46,820 participants with depression diagnose were further categorized into the treated and untreated groups. We compared the risks of dementia among different depression treatments groups in all participants that depressed as well as four courses of depressive symptoms by performing survival analyses.

**Result:**

Depression was associated with a 51% higher risk of dementia, among which the increasing, chronically high and chronically low courses were associated with increased dementia risk while no association was found in the decreasing course. Compare to those who were depressed but untreated, receiving depression treatments corresponded to a hazard ratio of 0.7 (95% confidence interval = 0.62‐0.77). Among the three detrimental courses, treatments for increasing and chronically low symptoms of depression were associated with a 42% and 29% lower risk of dementia while the reduction effect for chronically high symptoms was insignificant.

**Conclusion:**

The negative association between depression treatment and incident dementia was significant in the increasing and chronically low course, highlighting the necessity of timely interventional strategies before depression progress to a chronically severe state.

